# Inhibitory Effects of Dimeric Copper(II) bis(o-acetoxybenzoate) on Platelet-neutrophil adhesion and Thrombosis

**DOI:** 10.1155/MBD.2001.103

**Published:** 2001

**Authors:** Zhiqiang Shen, Lanou Wu, Weiping Liu, Jikai Liu, Zhihhe Chen

**Affiliations:** 1 Yunnan Pharmacological Laboratories of Natural Products Kunming Medical College Kunming 650031 China; 2 Kunming Institute of Precious Metals Kunming 650221 China; 3 Kunming Institute of Botany The Chinese Academy of Sciences Kunming 650204 China

## Abstract

Antithrombotic effect of the copper-aspirin complex (dimeric copper(II) bis(o-acetoxybenzoate) was evaluated in the model of venous thrombosis; its effects on platelet-neutrophil adhesion were investigated by use of rosette assay. The results showed that the intragastrically administered copper-aspirin complex (5, 7, and 10 mg kg^-1^) dose-dependently lowered the wet and dry thrombus weight; it significantly decreased the binding of arachidonic acid-activated platelets to neutrophils with an IC_50_ value of 41.5 μmol L^-1^. The results suggested that copper aspirinate inhibited platelet-neutrophil adhesion and resulted in a more potent antithrombotic activity.


### INHIBITORY EFFECTS OF DIMERIC COPPER(II) BIS(o-ACETOXYBENZOATE) ON PLATELET-NEUTROPHIL ADHESION AND THROMBOSIS[Δ]

Zhiqiang Shen*[1], Lanou Wu[1], Weiping Liu[2], Jikai Liu[3], and Zhihhe Chen[1]

[1]Yunnan Pharmacological Laboratories of Natural Products, Kunming Medical College, Kunming 650031 <szq2000@yahoo.com>
[2]Kunming Institute of Precious Metals, Kunming 650221, [3]Kunming Institute of Botany, The Chinese Academy of Sciences, Kunming 650204, China

**Abstract**

Antithrombotic effect of the copper-aspirin complex (dimeric copper(II) bis(o-acetoxybenzoate) was evaluated in the model of venous thrombosis; its effects on platelet-neutrophil adhesion were investigated by use of rosette assay. The results showed that the intragastrically administered copper-aspirin complex (5, 7, and 10 mg $kg^{-1}$) dose-dependently lowered the wet and dry thrombus weight; it significantly decreased the binding of arachidonic acid-activated platelets to neutrophils with an $IC_{50}$ value of 41.5 μmol $L^{-1}$. The results suggested that copper aspirinate inhibited platelet-neutrophil adhesion and resulted in a more potent antithrombotic activity.

**Key words** copper-aspirin complex, arachidonic acid, platelets, neutrophils, adhesion, venous thrombosis

### INTRODUCTION

Neutrophils and platelets, but not platelets alone, are closely associated with thromboembolic disorders [1]. The copper-aspirin complex was investigated to show lower gastrointestinal side effects [2] and more potent antiplatelet activity through elevating 6-keto-prostaglandin $F_{1\alpha}$ level while decreasing thromboxane $B_2$ generation in plasma[3]. The aim of this experiment is to further investigate the effect of copper aspirinate on platelet-neutrophil adhesion and its influence on ligated venous thrombosis.

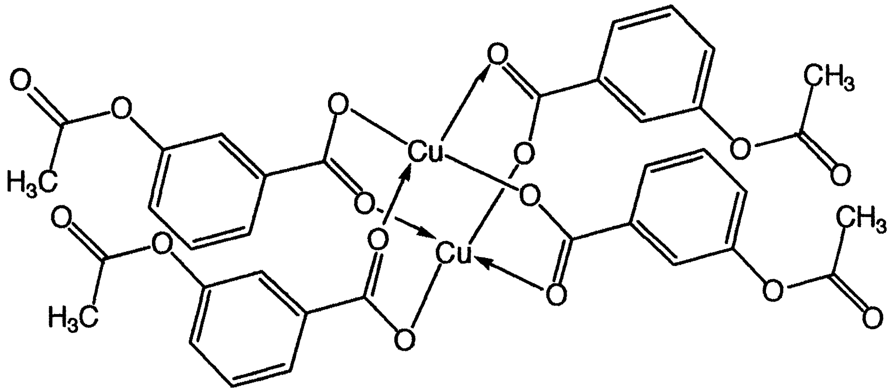

**Fig.** Structure of the copper-aspirin complex

### MATERIALS AND METHODS

### *Animals*

Male Sprague Dawley rats weighing 200 – 250 g were used in this study in accordance with the ethics committee of our Laboratories (Certificate 9805, the Administrative Commission of Medical Experimental Animals of Yunnan Public Health Bureau).

### *Chemicals and Drugs*

Copper aspirinate (Cu 14.99%, C 51.21%, and H 3.32%; purity > 98%) was synthesized by Kunming Institute of Precious Metals. It was dissolved in 0.9 % saline (pH 6.5). Crystalline aspirin was dissolved in 1% $Na_2CO_3$ before use. Arachidonic acid (AA) was from Sigma Chemical Co. It was dissolved in 100 mmol $L^{-1}$ $Na_2CO_3$ before use.

[Δ] This project was supported by Yunnan Natural Scientific Foundation, No: 98C066M

***Preparation of ligated venous thrombosis***

SD rats were divided into 5 groups with 10 rats each. A: 0.9 % saline as vehicle group; B: aspirin 20.0 mg / kg as reference; and C - E: 5, 7, and 10 mg / kg copper-aspirin complex treated groups. All the above substances were administered intragastrically 1 h before experiment. The method of Chen Changxun et al[4] was used to produce the model of venous thrombosis. Briefly, the rats were anesthetized by ip 30.0 mg / kg sodium pentobarbital. Then a midline incision of the abdomen was made and the inferior vena cava was isolated and ligated below the left renal vein level. The abdomen was then closed. One hour later, the abdomen was reopened. The thrombus in the inferior vena cava was collected into a glass dish for measurement of wet weight. It was then placed in a drying oven at 60°C for 20 h before measuring the dry weight. Significance was analyzed by *t* test.

***Preparation of platelets***

Blood sample from rat carotid artery was collected into plastic tubes, anticoagulated with 2.7 % EDTA. This sample was spun for 10 min at 180 μg to obtain platelet-rich plasma (PRP). PRP was further spun to pellet platelets at 1000 μg for 10 min. Platelet pellets were washed three times and resuspended in phosphate buffer solution (PBS, containing 1.0 % bovine serum albumin and 1.4 mmol $L^{-1}$ EDTA). Cell viability by Typan blue exclusion was above 95 % and cell counter was adjusted to $10^{11}$ cell $L^{-1}$.

***Preparation of neutrophils***

Neutrophils were isolated from the resulting blood by dextran sedimentation and followed by Ficoll-Hypaque (special gravity 1.077) and hypotonic lysis of erythrocytes. The cell pellet was resuspended in an erythrocyte lysis buffer composed of 155 mmol $L^{-1}$ $NH_4Cl$, 2.96 mmol $L^{-1}$ $KHCO_3$, and 3.72 mmol $L^{-1}$ EDTA. The tube was gently inverted and after 5 min the suspension was centrifuged at 350 μg for 10 min, and the cell pellet was washed in PBS lacking calcium; then resuspended in Hanks' solution (containing 1 mmol $L^{-1}$ $CaCl_2$ or 5 mmol $L^{-1}$ EGTA in vehicle, reflecting the situation with or without external calcium). Cells were adjusted to a count of $2 \times 10^6$ cell $mL^{-1}$. Cells prepared in this manner contained 98 % neutrophils and were 96 % viable.

***Rosette assay***

The method of Hamburger et al [5] was modified. Briefly, 50 μL aliquots of platelet suspension were placed in microtiter wells and exposed to arachidonic acid (0.1 mmol $L^{-1}$) for 15 min at room temperature without stirring. Fifty μL of 0.9 % saline or drug solution was added and incubated for 15 min at 37°C. Then 100 μL of neutrophils was added to the platelet suspension and incubated for 30 min at 4°C under rocking condition. One hundred neutrophils were scored for the presence (two or more platelets per neutrophil) or absence (zero or one platelet per neutrophil) of platelets. Neutrophils bearing two or more platelets were thus defined as rosettes. For each assay, done in triplicate, the rosetting score was assessed by two different observers.

### RESULTS AND DISCUSSION

In vehicle group, the wet and dry thrombus weight were 9.9 ± 1.8 and 4.1 ± 0.5 mg, respectively. Intragastric administration of aspirin 20 mg / kg significantly reduced the wet and dry thrombus weight to $2.2 \pm 1.3^{**}$ and $1.0 \pm 0.5^{**}$ mg ($^{**}$ $P < 0.01$ *vs* vehicle). The copper-aspirin complex administered intragastrically at 5, 7, and 10 mg / kg markedly decreased the wet thrombus weight to $4.9 \pm 2.1^{**}$, $2.7 \pm 1.9^{**}$, and $2.3 \pm 1.0^{**}$ mg, and suppressed the dry thrombus weight to $1.5 \pm 0.6^{**}$, $1.2 \pm 0.7^{**}$, and $0.8 \pm 0.6^{**}$ mg ($^{**}$ $P < 0.01$ *vs* vehicle), respectively. (Table I)

**Table I. Effect of the copper-aspirin complex administered intragastrically on rat venous thrombosis ( $n = 10, \bar{x} \pm s$, $^{**}$ $P < 0.01$ *vs* saline)**

| Drug | Dose (mg / kg) | Thrombus weight (mg) | |
|---|---|---|---|
| | | Wet | Dry |
| 0.9 % saline | — | 9.9 ± 1.8 | 4.1 ± 0.5 |
| Copper | 5 | $4.9 \pm 2.1^{**}$ | $1.5 \pm 0.6^{**}$ |
| Copper-aspirin complex | | | |
| | 7 | $2.7 \pm 1.9^{**}$ | $1.2 \pm 0.7^{**}$ |
| | 10 | $2.3 \pm 1.0^{**}$ | $0.8 \pm 0.6^{**}$ |
| Aspirin | 20 | $2.2 \pm 1.3^{**}$ | $1.0 \pm 0.5^{**}$ |

Our previous research indicated that the copper aspirin complex had a more potent antiplatelet activity [3]. But all this work was focused on platelets alone, not involved in neutrophils.

Platelet-neutrophil adhesion may play key factors in thromboembolic processes and adhesion of these two kinds of blood cells is involved in the process of thrombomodulation [1]. Activation of platelets increases neutrophil adhesion to foreign surfaces, neutrophile aggregation, lysosomal enzyme release, etc. Platelet-derived products are able to promote neutrophil chemotaxsis, enzyme release, and phagocytosis and to inhibit oxidative burst [6]. On the other hand, neutrophil-derived products can enhance platelet aggregation, serotonin release and cytoplasmic $Ca^{2+}$ movement [7]. It is necessary, therefore, to study an antithrombotic drug based on its influence on multiple cellular interactions.

In venous thrombosis, the copper-aspirin complex showed a dose-dependently inhibition. seven mg $kg^{-1}$ of copper aspirinate obtained nearly equal antithrombotic effect to 20 mg $kg^{-1}$ of its parent compound—aspirin. Obviously, copper aspirinate exhibited a more potent antithrombosis.

The percentage of rosettes in vehicle was 71.8 or 11.6 % in a condition of external 1 mmol $L^{-1}$ $CaCl_2$ or 5 mmol $L^{-1}$ EGTA. Copper aspirinate and aspirin significantly decreased the binding of platelets to neutrophils with 1 mmol $L^{-1}$ external $Ca^{2+}$, giving $IC_{50}$ values 41.5 and 51.4 μmol $L^{-1}$, respectively (Table II).

**Table II. Effect of the copper-aspirin complex on the binding of arachidonic acid-stimulated platelets to neutrophils in rats ($n = 9, x \pm s$, $^{**}P < 0.01$ *vs* 0.9 % saline)**

| Drug / μmol $L^{-1}$ | Adhesion (%) | |
|---|---|---|
| | Copper-aspirin complex | Aspirin |
| 0 | 71.9 ± 5.2 | 71.9 ± 5.2 |
| 15 | 58.9 ± 5.1 | 66.9 ± 3.9 |
| 30 | 40.7 ± 3.7 | 46.0 ± 4.7 |
| 60 | 30.1 ± 2.3 | 29.0 ± 1.2 |
| 120 | 24.0 ± 2.1 | 25.2 ± 2.4 |

Thrombus formation is mediated by the platelet-neutrophil interactions including cell binding and platelet aggregation. In vehicle (0.9 % saline) containing external 1 mmol $L^{-1}$ $CaCl_2$ or 5 mmol $L^{-1}$ EGTA, the percentage of rosettes induced by AA was 71.8 or 11.2 %, demonstrating a calcium dependent relationship between platelet-neutrophil adhesion. Both the copper-aspirin complex and aspirin showed significant inhibition on rosetting between AA-activated platelets and neutrophils, and was more active than aspirin based on the $IC_{50}$ value. It is suggested that the copper-aspirin complex showed more potent antithrombotic activity than aspirin due to its higher activity on platelet-neutrophil adhesion.

In conclusion, the copper-aspirin complex may be more potential in treating thromboembolic diseases because of its lower gastrointestinal side effects.

### REFERENCES

1. Marcus A. J., Safier L. B. *FASEB. J.*, 1993, **7**, *516*-522
2. Li L., Wu L. O., Liu W. P., Chen Z. H. *J. Kunming Med. Coll.*, 1996, **17**, 1-3
3. Shen Z. Q., Li L., Wu L. O., Liu W. P., Chen Z. H. *Platelets*, 1999, **10**, 345-348.
4. Chen C. X., Jin R. M., Li Y. K., Zhong J., Yue,L., Chen S. C., Zhong J. Y.*Acta. Pharmacol. Sin*, 1992, **13**, 126-130.
5. Hambueger S. A., McEver R. P. *Blood*, 1990, **75**, 550-554
6. Weksler B. B. In: Gallin, J. I., Golstein, I. M., Synderman, R. (eds) *Platelets*. Raven, New York, 1988, pp. 543-557
7. Maschio A. D., Evangelista V., Rajtar G., Chen Z. M., Cerletti C., Gaetano G. *Am. J. Physiol. ,1990*, **258**, H870-H879

**Received: April 24, 2001 – Accepted: May 2, 2001 –**
**Accepted in publishable format: May 10, 2001**

